# Serum Calcification Propensity T50 Is Associated with Soluble Thrombomodulin in Patients on Hemodialysis

**DOI:** 10.3390/jcm13123491

**Published:** 2024-06-14

**Authors:** Takeshi Tadokoro, Akihiko Kato, Hiromitsu Ohmori, Tomio Matsumoto, Makoto Kuro-O, Tsuyoshi Kobayashi, Hideki Ohdan

**Affiliations:** 1 Department of Surgery, National Hospital Organization Yanai Medical Center, 95 Ihonosho, Yanai-shi 742-1352, Yamaguchi, Japan; tomio0102@outlook.jp; 2 Department of Gastroenterological and Transplant Surgery, Graduate School of Biomedical and Health Science, Hiroshima University, 1-2-3 Kasumi, Minami-ku, Hiroshima City 734-8551, Hiroshima, Japan; tsukoba@hiroshima-u.ac.jp (T.K.); hohdan@hiroshima-u.ac.jp (H.O.); 3 Blood Purification Unit, Hamamatsu University Hospital, 1-20-1 Handayama, Chūō-ku, Hamamatsu 431-3192, Shizuoka, Japan; a.kato@hama-med.ac.jp; 4 Department of Pediatrics, National Hospital Organization Yanai Medical Center, 95 Ihonosho, Yanai-shi 742-1352, Yamaguchi, Japan; yhohmori18252922@icloud.com; 5 Division of Anti-aging Medicine, Center for Molecular Medicine, Jichi Medical University, 3311-1 Yakushiji, Shimotsuke 329-0498, Tochigi, Japan; mkuroo@jichi.ac.jp

**Keywords:** soluble thrombomodulin, T50, calcification propensity, coronary artery calcification, coronary artery disease, hemodialysis

## Abstract

**Background/Objectives:** Levels of circulating soluble thrombomodulin (sTM), an anticoagulant factor, are associated with the severity and progression of arteriosclerotic diseases. However, the role of elevated sTM levels remains to be clarified in patients on dialysis. As the calcification propensity time T50 is a novel marker of arterial calcification, we aimed to determine the association between sTM and T50 in patients on hemodialysis (HD). **Methods:** This cross-sectional study included 49 adult patients on maintenance HD. Correlation analysis was performed to test the association between T50 and patient characteristics. Linear regression was used to evaluate the association between T50 and sTM. **Results:** Partial correlation analysis showed a strong association between T50 and glycated albumin, phosphorous, and sTM levels (partial correlation coefficient: r [partial] = −0.359, *p* = 0.023; r [partial] = −0.579, *p* < 0.001; and r [partial] = 0.346, *p* = 0.029, respectively). Multivariate linear regression analysis revealed that only sTM level was significantly and positively associated with T50 (β = 0.288; t = 2.27; *p* = 0.029; 95% confidence interval, 0.082–1.403). **Conclusions:** sTM is independently and positively associated with the propensity time for calcification, suggesting that sTM could be a good marker of arterial calcification progression in patients on HD.

## 1. Introduction

Thrombomodulin (TM) is a glycosylated type I transmembrane molecule composed of 557 amino acids with a molecular weight of approximately 75 kDa. TM does not possess intrinsic enzymatic activity that is expressed in endothelial cells. Approximately half of the extracellular domain consists of N-terminal globular domain, whereas the remainder of the extracellular portion of TM is composed of an extended stalk of six epidermal growth factor modules [1,2]. TM is also a cofactor for the thrombin-catalyzed activation of protein C, which exerts anticoagulant effects. Additionally, TM exerts anti-inflammatory effects by degrading high mobility group box 1 [3]. Soluble TM (sTM), which comprises several TM domains, is the major circulating TM generated by either enzymatic or chemical cleavage. sTM circulates in the blood at concentrations of 3–50 ng/mL. However, the role of circulating TM for vascular physiology and pathophysiology remains unclear [4,5]. Increased plasma sTM levels are found in patients with disseminated intravascular coagulation, sepsis, and pre-eclampsia; therefore, sTM is established as a biomarker of endothelial dysfunction [6,7,8].

Endothelial dysfunction is a crucial component of cardiovascular disease (CVD). In many cases, TM shedding from the endothelial cells of arteries and veins contributes to elevated levels of circulating sTM. Therefore, circulating sTM levels are closely related to CVD severity, including abdominal aortic aneurysm, acute myocardial infarction, and atherosclerosis [5]. Salomaa et al. [9] found that patients with increased plasma sTM levels had a higher chance of developing carotid atherosclerosis in a large cohort study [9]. Plasma sTM levels could be used as a biomarker to predict the development of clinical events in diabetes patients with ischemic heart disease [10]. In patients with chronic kidney disease (CKD), circulating sTM levels increase proportionally with the advancement of CKD stage, as sTM is primarily excreted by the kidneys [11]. However, there have been few studies testing the association of elevated sTM with vascular damage.

Recently, the serum calcification propensity test (also known as T50), primary-to-secondary calciprotein particle (CPP) transformation time, has been proposed to quantify serum anti-calcification buffer capacity. CPP is a colloidal mineral–protein complex mainly composed of solid-phase calcium (Ca), phosphorous (P), and serum protein fetuin-A. Tiny amorphous Ca-P precipitates in the solution agglomerate initially and subsequently transit to the crystalline phase, leading to the maturation of primary and secondary CPPs [12]. T50 reflects the endogenous ability of serum to prevent Ca-P precipitation [13]. A shorter time of T50 is considered a reflection of the increased calcification propensity of serum, whereas a longer time reflects the greater resistance of serum to calcification.

In patients with CKD, T50 is associated with the progression of coronary arterial calcification, incident cardiovascular events, and all-cause mortality [13]. A shorter time of T50 is also an independent predictor of death after CAD events in patients on hemodialysis (HD) [14]. Therefore, T50 may be useful to assess the severity of vascular calcification in patients with HD. Furthermore, a therapeutic approach to increase T50 may be effective in mitigating clinical complications related to vascular calcification [15].

In the present study, we aimed to evaluate the association between sTM and T50 to examine the role of elevated serum sTM levels on vascular calcification in patients on HD.

## 2. Materials and Methods

### 2.1. Study Design

This study comprised 49 adult patients on maintenance HD at the National Hospital Organization Yanai Medical Center (Yamaguchi, Japan). The patients were in stable condition, and none of them had been experiencing either advanced cancer, active collagen disease, or active infections. All patients underwent regular HD for 4–5 h, three times per week, at a blood flow rate of 230–360 mL/min with bicarbonate buffer dialysate. Detailed medical information about the present study was provided to the participants or proxies, and written consent was obtained for participation in this study. This study was approved by the Regional Ethical Review Board of the National Hospital Organization Yanai Medical Center (Y-5-1) and conducted in accordance with the World Medical Association’s Declaration of Helsinki guidelines. Subsequently, we conducted our study using anonymous clinical data under close supervision after receiving approval from the medical ethics committee of our hospital.

### 2.2. Data Collection

Blood samples were collected from the arterial site of the arteriovenous fistula at the start of each dialysis session. Kt/V was calculated using a single-pool urea kinetic model [16]. Basal biochemical parameters were measured using standard laboratory techniques. We collected the serum Ca level using the following formula: corrected Ca = serum Ca + (4 − serum albumin [Alb]). Glycated Alb (GA) and trace elements were measured at Bio-Medical Laboratories (Tokyo, Japan).

### 2.3. Measurement of sTM Levels

sTM levels were measured using commercially available assay kits based on a chemiluminescent enzyme immunoassay (STACIA CLEIA^TM^ [normal range, 12.1–24.9 U/mL]; PHC Holdings Corporation, Tokyo, Japan). In brief, 60 μL of magnetic latex reagent was added to 5 μL of specimen, warmed at 37 °C for 6.2 min, subjected to bf separation, and washed. Next, 100 μL of enzyme-labeled antibody reagent was added, warmed at 37 °C for 4.4 min, subjected to bf separation, and washed, followed by the addition of 100 μL of substrate solution. Luminescence was measured after 2.7 min of reaction at 37 °C. Luminescence was compared to that of a standard solution used in the same manner to determine the concentration of sTM in the sample.

### 2.4. Measurement of T50

T50 was quantified according to a method originally developed by Pasch et al. [17]. Serum T50 levels were measured at the Division of Anti-Aging Medicine, Center for Molecular Medicine, Jichi Medical University (Tochigi, Japan). Briefly, the addition of 10 mM Ca and 6 mM P to serum samples triggered the formation of primary CPPs. The primary CPPs included fetuin A, Alb, and amorphous Ca P. Primary CPPs spontaneously transformed into secondary CPPs. To detect the transformation, time to a rapid increase in turbidity during a thermo-constant incubation at 37 °C was quantified via time-resolved nephelometry. The one-half transformation time was determined as the calcification propensity specific to the individual serum samples.

### 2.5. Statistical Analyses

Continuous variables are expressed as means ± standard deviation or medians (interquartile ranges). Categorical variables are expressed as numbers and percentages. For descriptive and comparative purposes, baseline patient characteristics were stratified according to T50 tertiles. Means or medians were compared between the T50 tertile groups using analysis of variance, Mann–Whitney U test, or Kruskal–Wallis test, as appropriate. Categorical variables were compared using the χ^2^ or Fisher’s exact test. Correlation analysis was performed to investigate the association between T50 and variables. Partial correlation was used to adjust for the associated variables (age; Kt/V; low-density lipoprotein-cholesterol [LDL-C], GA, Alb, corrected Ca, P, and magnesium [Mg] levels; prothrombin time [PT]; activated partial thromboplastin time [APTT]; antithrombin [AT] activity; and sTM levels). A multivariate linear regression analysis was performed to investigate the association between the dependent variable and multiple independent covariates. Independent variables were selected based on the results of the univariate analysis for which p values were *p* < 0.05. The regression models were assessed for the absence of multicollinearity. Statistical analyses and figure drawings were performed using JMP Pro (version 17; SAS Institute, Cary, NC, USA). Statistical significance was set at *p* < 0.05.

## 3. Results

The median age of the participants was 75.0 (70.0–81.5) years, and 57% of the patients were male. HD vintage was 34.0 (16.5–44.5) months. The median of T50 was 112.5 (91.4–153.1). Because serum T50 values were not symmetrically distributed in this study (Shapiro–Wilk test, *p* = 0.09, skewness = 0.54; Figure S1), we classified all patients into three tertiles according to their T50, starting with the middle tertile ranging from 95 to 135 min. The basal characteristics of the overall and the classified patients according to the three tertiles of T50 are presented in Table 1.

### 3.1. Association between Clinical Parameters and T50

There was a significant and inverse association between T50 and age (r = −0.283, *p* = 0.049) and GA level (r = −0.330, *p* = 0.02) (Figure S2A,D). LDL-C level was positively correlated with T50 (r = 0.372, *p* = 0.009) (Figure S2C). We also found significant differences among the T50 tertiles for Kt/V (*p* = 0.047, Table 1); however, there was no association between Kt/V and T50 (r = 0.209, *p* = 0.149, Figure S2B).

Significant differences in serum P (*p* < 0.001) and corrected Ca (*p* = 0.031) levels were also observed among the T50 tertiles (Table 1). Additionally, both corrected Ca (r = −0.408, *p* = 0.004) and serum P (r = −0.283, *p* = 0.049) levels were significantly and inversely correlated with T50 (Figure S2F,G). In contrast, there was no association between serum Alb (r = 0.262, *p* = 0.069) and Mg (r = 0.136, *p* = 0.353) levels and T50 (Figure S2E,H).

### 3.2. Association between Coagulation Parameters and T50

The mean sTM level was 67.1 (51.0–77.9) U/mL, and the AT activity was 76.0 (66.0–88.5)%.

Serum sTM levels significantly correlated with T50 (r = 0.328, *p* = 0.022) (Figure S2L). In patients with the highest tertiles of T50, sTM levels tended to be higher (*p* = 0.071). AT activity was significantly higher in the top tertile compared with that in the other two tertiles (*p* = 0.002, Table 1). A significant and inverse correlation was found between AT activity and T50 (r = 0.525, *p* < 0.001) (Figure S2J). Both PT (r = −0.311, *p* = 0.030) and APTT (r = −0.427, *p* = 0.002) were significantly and inversely correlated with T50 (Figure S2I,K).

### 3.3. Partial Correlation Diagram

**Figure 1:**
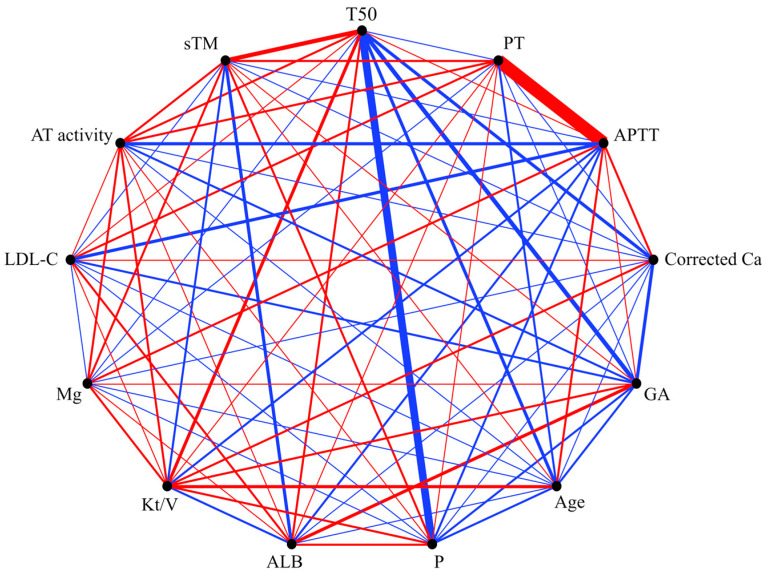
Partial correlation diagram between baseline patient characteristics and serum T50. The color of the line shows a positive (red) or negative (blue) partial correlation between the variables. The width of the line indicates the strength of the partial correlation between the variables. The thicker the line, the stronger the partial correlation. Alb, albumin; GA, glycated albumin; P, phosphate; Ca, calcium; Mg, magnesium; LDL-C, low-density lipoprotein-cholesterol; sTM, soluble thrombomodulin; AT, antithrombin; PT, prothrombin time; APTT, activated partial thromboplastin time.

We used a partial correlation diagram to explore the associations and potential causal associations between the variables (Figure 1).

There was a correlation between two variables, including T50, which was adjusted for all other variables. Consequently, T50 was significantly correlated with serum GA (r [partial] = −0.359, *p* = 0.023), P (r [partial] = −0.579, *p* < 0.001), and sTM (r [partial] = 0.346, *p* = 0.029) levels (Table 2).

### 3.4. Independent Factors Affecting T50

To determine the independent factors affecting T50, we performed univariate and multivariate linear regression analyses (Table 3).

In the univariate analysis, T50 was significantly associated with age; LDL-C, C-reactive protein (CRP), and GA levels; PT; APTT; sTM level; and AT activity. In the multiple regression analysis, only sTM (β = 0.288; t = 2.27; *p* = 0.029; 95% confidence interval, 0.082–1.403) became a significant determinant of T50 following the adjustment for LDL-C, CRP, and GA levels; APTT; and AT activity (Table 3).

## 4. Discussion

To the best of our best knowledge, this is the first study to examine an association between the calcification propensity time (T50) and coagulation parameters in patients on prevalent dialysis. In univariate analyses, T50 was positively correlated with sTM level and AT activity, whereas it was negatively correlated with PT and APTT. However, multivariate regression analysis revealed that only sTM became an independent determinant of T50.

TM can promote apoptosis and vascular calcification in cultured vascular smooth muscle cells [18]. A recent in vitro study demonstrated that secondary CPPs directly caused endothelial cell dysfunction by impairing nitric oxide metabolism [19]. Because an elevated sTM level is positively associated with advanced radial artery calcifications in patients on HD [20], we evaluated the association between sTM and T50, a marker for CPP formation.

In the present study, increased sTM level was positively associated with prolonged T50. Although TM is a thrombin receptor on endothelial cells that is involved in promoting activation of the anticoagulant protein C pathway during blood coagulation, TM also exerts protective anti-inflammatory properties. An experimental study demonstrated that recombinant human sTM exerted anti-inflammatory effects by inhibiting the rolling adhesion of neutrophils to vascular endothelial cells in mice [21]. Recombinant sTM also mitigates coronary arteritis in a mouse model of vasculitis [22]. It was found that individuals with a high level of sTM were associated with a significant reduction in the relative risk of coronary heart disease events [9]. Conversely, sTM did not predict future coronary events in apparently healthy, middle-aged patients in another large prospective case–cohort study [23]. Therefore, serum sTM measurement may be useful for monitoring the severity of vascular calcification rather than predicting future CVD events in clinical settings. Further studies are required to explore the intricate role of sTM in the pathophysiology of arteriosclerotic disease in patients on dialysis.

Warfarin, a vitamin K antagonist, increases arterial calcification, including coronary and peripheral vessels. This enhanced vascular calcification is mainly caused by the inhibition of the enzyme matrix gamma-carboxyglutamate Gla protein [24]. We found that administration of vitamin K antagonist was more frequent in patients with the the lowest tertile of T50 (Table 1). In addition, prolonged PT was inversely correlated with a shorter T50. Kapustin et al. [25] showed that prothrombin, a vitamin K-dependent coagulation factor, could inhibit exosome-mediated calcification in vascular smooth muscle cells. However, recent randomized controlled trials demonstrated no association between vitamin K supplementation and serum calcification propensity [26,27]. In patients on HD, 1 year of vitamin K supplementation also did not change the blood levels of the PT fragment [28]. Therefore, it is likely that vitamin K deficiency plays a minor role in CPP formation in patients on dialysis.

In this study, we found that GA level was negatively associated with T50. In patients on dialysis, GA is a more sensitive marker of short-term glycemic control compared with hemoglobin (Hb) A1c owing to uremia-induced shorter red blood cell half-life [29]. As HbA1c level was inversely associated with T50 in patients with type 2 diabetes [30], our data support the hypothesis that poor glycemic control may promote vascular calcification.

This study has some limitations. First, the sample size was small, which prevented the identification of several relevant factors. Second, as our study was a monocentric cross-sectional study, we could not establish a causal association between sTM level and T50. Finally, we performed a single T50 measurement and did not analyze longitudinal changes. Previous studies have suggested that longitudinal changes in T50 may offer better prognostic value compared with single T50 measurement [31]. Thus, longitudinal observation will be required to determine the role of sTM for vascular calcification.

## 5. Conclusions

In this cross-sectional study, we demonstrated that sTM level was independently associated with vascular calcification propensity. High levels of sTM were correlated with low T50, suggesting that sTM could be a good marker of CPP formation in patients on dialysis. Further prospective studies will be required to clarify the role of sTM on vascular calcification propensity in patients on dialysis.

## Figures and Tables

**Table 1:** Baseline characteristics stratified according to the T50 tertiles.

Variables	Units	Overall	Tertile 1	Tertile 2	Tertile 3	*p* Value
			(<95 min)	(95–135 min)	(>135 min)	
		(n = 49)	(n = 16)	(n = 17)	(n = 16)	
Age	Years	75.0 (70.0–81.5)	74.5 (73.0–82.0)	78.0 (69.5–85.0)	74.0 (69.0–79.8)	0.615
Sex, male	n (%)	28 (57.1)	10 (62.5)	10 (58.8)	8 (50.0)	0.763
BMI	kg/m^2^	20.6 (18.0–22.9)	18.3 (16.9–21.8)	21.0 (18.1–23.2)	21.2 (19.9–25.4)	0.467
CAD	n (%)	14 (28.6)	5 (31.3)	5 (29.4)	4 (25.0)	0.922
Atrial fibrillation	n (%)	5 (10.2)	3 (18.8)	1 (5.9)	1 (6.3)	0.388
Hemodialysis vintage	Month	34.0 (16.5–44.5)	22.0 (11.5–39.8)	32.0 (14.0–49.5)	40.0 (26.5–47.8)	0.609
Kt/V		2.1 (1.8–2.5)	2.1 (1.8–2.5)	1.9 (1.7–2.3)	2.4 (2.0–2.7)	0.047
Hb	g/dL	11.2 (9.8–12.0)	11.9 (10.3–12.6)	10.7 (9.4–11.4)	11.2 (9.4–12.0)	0.274
Alb	g/L	3.0 (2.6–3.2)	3.0 (2.5–3.3)	2.9 (2.5–3.2)	3.0 (2.7–3.2)	0.789
TG	mg/dL	104.0 (81.5–138.0)	93.0 (78.2–119.0)	99.0 (77.5–126.0)	118.0 (95.3–180.8)	0.407
LDL-C	mg/dL	78.0 (48.5–102.5)	71.5 (42.5–80.8)	78.0 (45.0–103.0)	89.5 (66.3–109.8)	0.201
CRP	mg/dL	0.3 (0.1–0.9)	0.3 (0.1–1.6)	0.5 (0.2–1.0)	0.2 (0.1–0.5)	0.162
GA	%	17.6 (14.7–21.4)	17.6 (15.2–24.0)	19.3 (15.4–21.6)	15.8 (13.1–19.0)	0.095
Corrected Ca	mg/dL	9.6 (9.1–10.2)	10.1 (9.1–10.7)	9.6 (9.4–10.2)	9.5 (8.9–9.8)	0.031
P	mmol/L	4.2 (3.1–4.8)	4.8 (4.5–6.2)	3.7 (2.6–4.4)	3.8 (3.0–4.5)	<0.001
Mg	mg/dL	2.6 (2.2–2.8)	2.6 (2.5–2.7)	2.2 (2.1–2.8)	2.6 (2.1–3.0)	0.339
Zn	mg/dL	52.0 (43.5–57.0)	52.0 (41.3–57.0)	53.0 (44.5–59.0)	48.0 (43.3–55.0)	0.697
PT	Seconds	12.0 (11.5–12.9)	12.9 (11.1–14.6)	12.1 (11.8–12.8)	11.9 (11.4–12.2)	0.077
APTT	Seconds	35.0 (30.7–41.7)	36.3 (31.1–45.3)	36.3 (32.3–46.6)	31.3 (28.6–36.1)	0.048
D-dimer	μg/mL	1.6 (1.3–2.6)	1.7 (1.2–3.3)	1.6 (1.2–2.5)	1.4 (1.2–2.7)	0.844
sTM	U/mL	67.1 (51.0–77.9)	65.5 (47.4–79.3)	57.1 (48.4–72.2)	73.5 (63.5–78.6)	0.071
AT activity	%	76.0 (66.0–88.5)	76.0 (66.5–79.8)	69.0 (62.0–77.0)	88.5 (73.4–95.8)	0.002
Medications						
Magnesium oxide	n (%)	9 (18.4)	1 (6.3)	5 (29.4)	3 (18.8)	0.229
Statins	n (%)	10 (20.4)	2 (12.5)	3 (17.7)	5 (31.3)	0.396
Vitamin D analogs	n (%)	13 (26.5)	2 (12.5)	5 (29.4)	6 (37.5)	0.262
Phosphate binders	n (%)	15 (30.6)	5 (31.3)	4 (23.5)	6 (37.5)	0.683
Vitamin K antagonists	n (%)	4 (8.2)	4 (25.0)	0 (0)	0 (0)	0.011
Antiplatelet drugs	n (%)	4 (8.2)	0 (0)	3 (17.7)	1 (6.3)	0.17
ARB	n (%)	9 (18.4)	5 (31.3)	3 (17.7)	1 (6.3)	0.188

BMI, body mass index; CAD, coronary artery disease; Hb, hemoglobin; Alb, albumin; TG, triglyceride; LDL-C, low-density lipoprotein-cholesterol; CRP, C-reactive protein; GA, glycated albumin; Ca, calcium; P, phosphate; Mg, magnesium; Zn, zinc; PT, prothrombin time; APTT, activated partial thromboplastin time; sTM, soluble thrombomodulin; AT activity, antithrombin activity; ARB, angiotensin II receptor blocker.

**Table 2:** Partial correlation coefficients between baseline patient characteristics and serum T50.

	Partial Correlation Coefficient	*p* Value
Age	−0.257	0.109
Kt/V	0.266	0.097
GA	−0.359	0.023
Alb	−0.193	0.232
LDL-C	0.086	0.598
Corrected Ca	−0.291	0.069
P	−0.579	<0.001
Mg	−0.033	0.838
PT	−0.083	0.609
APTT	0.007	0.964
AT activity	0.208	0.198
sTM	0.346	0.029

GA, glycated albumin; Alb, albumin; LDL-C, low-density lipoprotein-cholesterol; Corrected Ca, corrected calcium; P, phosphate; Mg, magnesium; PT, prothrombin time; APTT, activated partial thromboplastin time; AT activity, antithrombin activity; sTM, soluble thrombomodulin.

**Table 3:** Univariate and multivariate linear regression analyses for the determinants of T50.

Variables	Univariate Analysis			Multivariate Analysis			
	*t*	*p*	95% CI	*β*	*t*	*p*	95% CI
Age	−2.02	0.049	−2.846 to −0.007				
Sex, female	0.08	0.936	−11.675–12.660				
Hemodialysis vintage	0.17	0.863	−0.367–0.436				
Kt/V	1.47	0.149	−7.087–45.188				
Hb	−0.26	0.796	−8.914–6.874				
Alb	1.86	0.069	−1.944–50.582				
LDL-C	2.74	0.009	0.131–0.852	0.166	1.14	0.261	−0.164–0.588
Mg	0.94	0.352	−14.263–39.233				
Zn	0.56	0.577	−0.742–1.317				
CRP	−2.4	0.021	−12.007 to −1.048	−0.221	−1.45	0.155	−10.382–1.711
GA	−2.4	0.021	−4.570 to −0.401	−0.209	−1.6	0.118	−3.424–0.401
PT	−2.24	0.03	−3.236 to −0.177				
APTT	−3.23	0.002	−2.426 to −0.565	−0.074	−0.41	0.683	−1.480–0.979
D-dimer	−0.93	0.359	−7.870–2.905				
sTM	2.38	0.022	0.135–1.621	0.288	2.27	0.029	0.082–1.403
AT activity	4.23	<0.001	0.787–2.212	0.169	1.01	0.319	−0.470–1.411

*t*, *t*-test statistic; β, standardized beta coefficient; CI, confidence interval, Hb, hemoglobin; Alb, albumin; LDL-C, low-density lipoprotein-cholesterol; Mg, magnesium; Zn, zinc; CRP, C-reactive protein; GA, glycated albumin; PT, prothrombin time; APTT, activated partial thromboplastin time; sTM, soluble thrombomodulin; AT activity, antithrombin activity.

## Data Availability

The data generated and analyzed in this study are available upon reasonable request.

## Supplementary Materials

The following supporting information can be downloaded at: https://www.mdpi.com/article/10.3390/jcm13123491/s1, Figure S1: Frequency distribution of serum T50; Figure S2: Correlation analysis between baseline patient characteristics and serum T50.
