# Supplementary material for: Serum Calcification Propensity T50 Is Associated with Soluble Thrombomodulin in Patients on Hemodialysis
Source: J Clin Med. 2024 Jun 14;13(12):3491. doi: 10.3390/jcm13123491 (PMC11205071; doi:10.3390/jcm13123491)
Supplement: Supplementary file 1 [file jcm-13-03491-s001.zip › jcm-3038995-supplementary.pptx]

## Slide 1
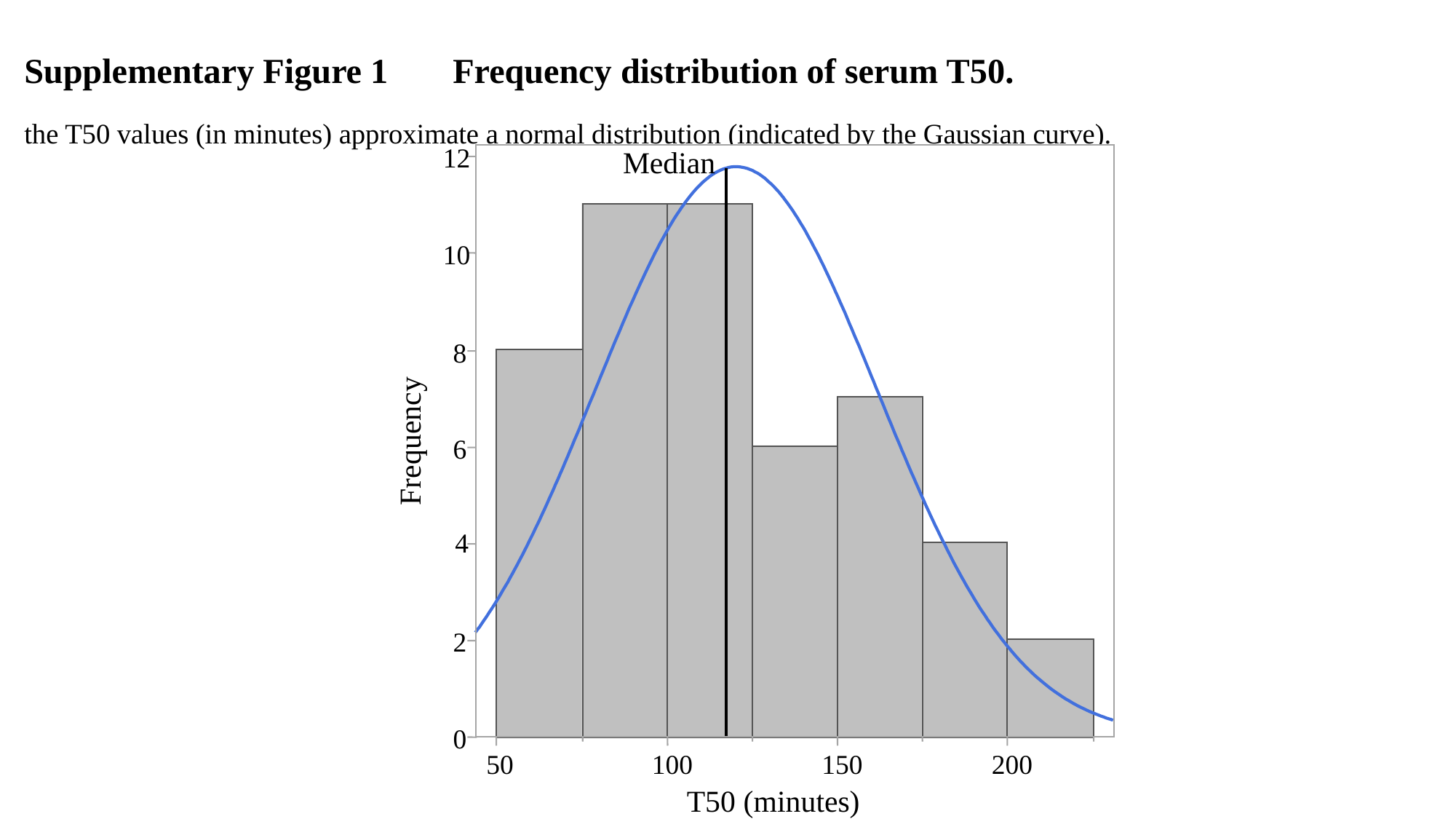

Supplementary Figure 1　Frequency distribution of serum T50.
the T50 values (in minutes) approximate a normal distribution (indicated by the Gaussian curve).
12
Median
10
8
Frequency
6
4
2
0
50
100
150
200
T50 (minutes)

## Slide 2
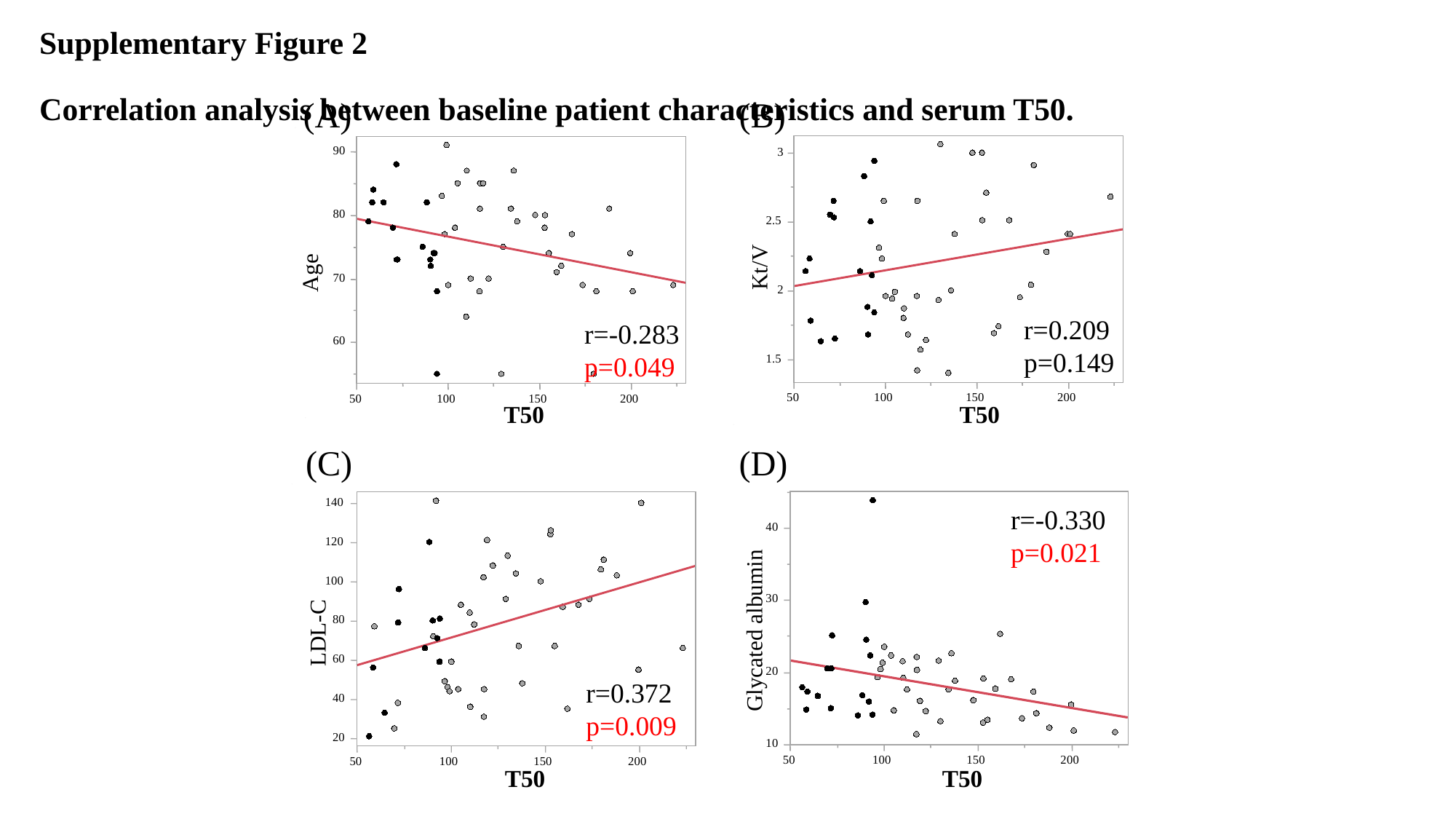

Supplementary Figure 2
Correlation analysis between baseline patient characteristics and serum T50.
(A)
(B)
90
3
80
2.5
Kt/V
Age
70
2
r=0.209
p=0.149
r=-0.283
p=0.049
60
1.5
50
100
150
200
50
100
150
200
T50
T50
(D)
(C)
140
r=-0.330
p=0.021
40
120
100
30
Glycated albumin
80
LDL-C
60
20
r=0.372
p=0.009
40
20
10
50
100
150
200
50
100
150
200
T50
T50

## Slide 3
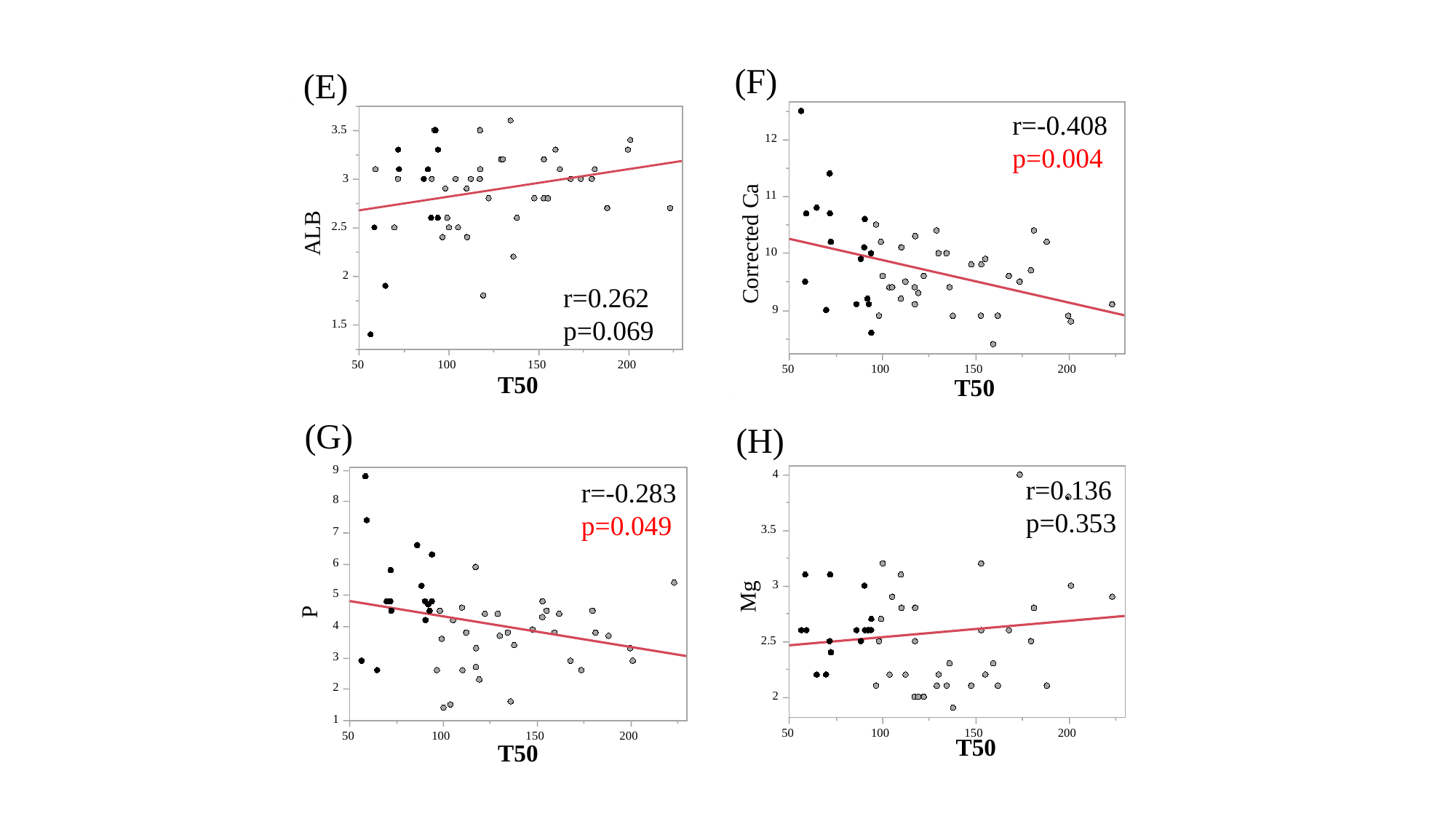

(F)
(E)
r=-0.408
p=0.004
3.5
12
3
11
2.5
ALB
Corrected Ca
10
2
r=0.262
p=0.069
9
1.5
50
100
150
200
50
100
150
200
T50
T50
(G)
(H)
9
4
r=0.136
p=0.353
r=-0.283
p=0.049
8
3.5
7
6
3
Mg
5
P
4
2.5
3
2
2
1
50
100
150
200
50
100
150
200
T50
T50

## Slide 4
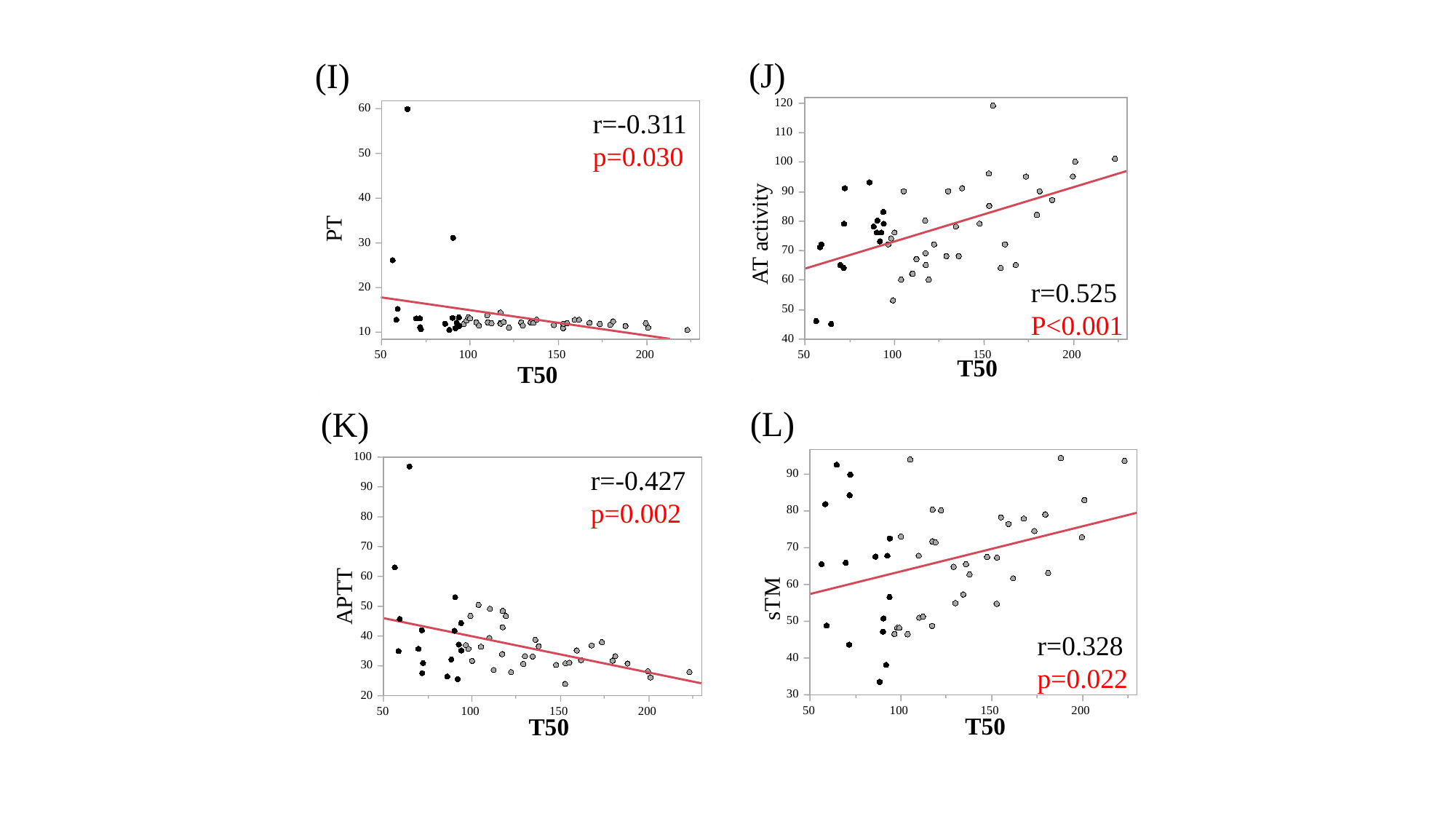

(J)
(I)
120
60
r=-0.311
p=0.030
110
50
100
90
40
80
AT activity
PT
30
70
r=0.525
P<0.001
60
20
50
10
40
50
100
150
200
50
100
150
200
T50
T50
(L)
(K)
100
r=-0.427
p=0.002
90
90
80
80
70
70
60
60
APTT
sTM
50
50
r=0.328
p=0.022
40
40
30
30
20
50
100
150
200
50
100
150
200
T50
T50
